# Removal of Silicon from Magnesite by Flotation: Influence of Particle Size and Mechanical Mechanism

**DOI:** 10.3390/ma16186095

**Published:** 2023-09-06

**Authors:** Ruinan Zhang, Zhaoyang Liu, Jingkun Yu

**Affiliations:** 1 School of Metallurgy, Northeastern University, Shenyang 110819, Chinayujk@smm.neu.edu.cn (J.Y.); 2 Key Laboratory for Ecological Metallurgy of Multimetallic Mineral, Ministry of Education, Shenyang 110819, China

**Keywords:** magnesite, microfine particle flotation, particle agglomeration, mechanism

## Abstract

The feasibility of producing high-density sintered magnesia with a one-step sintering method was investigated by utilizing finely ground magnesite as raw materials for direct flotation. The mechanism of flotation desilication of microfine grain magnesite was investigated by combining microstructure and chemical properties. The results showed that dodecylamine (DDA) has a sorting effect on magnesite reverse flotation desilication. Under the premise of 150 mg/L sodium polyacrylate (PAANa) as an inhibitor and 300 mg/L DDA as a collector, the content and recovery rate of MgO can reach 83.91% and 78.78%, respectively. When sodium oleate (NaOL) was used as a collector, the recovery rate of MgO was only 49.22%; therefore, it is unsuitable for magnesite purification. The flotation effect was affected because MgO particles and SiO_2_ particles agglomerated in the flotation process. The flotation agent cannot work for a single element but works for the mineral agglomerate. While collecting Si elements, the agglomerated MgO was also brought into the froth layer, making flotation impossible.

## 1. Introduction

China is the leading producer and exporter of magnesia refractories and has the richest magnesite reserves in the world [1,2]. However, due to over-exploitation and rising demand for magnesium products, high-grade magnesite reserves are depleting, and the ore dilution has changed seriously year by year. Consequently, the exploitation of low-grade magnesite is becoming necessary [3].

In general, magnesite is often found alongside gangue minerals, such as quartz and dolomite [4,5]. Various techniques, including flotation separation, magnetic separation, thermal separation, chemical beneficiation, and electrical separation, are used to purify magnesite [6]. Among these techniques, froth flotation is the most commonly used [7,8]. It uses a collector to increase the quality of the ore and reduce impurities in the concentrate by exploiting the differences in the physical and chemical characteristics of the mineral surfaces [9,10]. Hydrophobic particles are made to attach to bubbles, and the value mineral concentrate is carried to the froth phase and recovered, while hydrophilic particles, typically as gangue minerals, are kept in the pulp phase as tailings [11,12]. The above reaction process is for direct flotation, while reverse flotation is the opposite [13].

The main useful component in magnesite ore is magnesite, and the gangue minerals are mainly talc, quartz, and dolomite, especially quartz, which is one of the main factors affecting the sintering performance of magnesite. In order to separate quartz from magnesite and obtain more ideal flotation results, scientists have conducted systematic research on flotation methods, reagents, etc. [14]. Gence et al. [15] studied the adsorption mechanism of sodium oleate in magnesite flotation, demonstrating the feasibility of sodium oleate flotation. Feng Qigui et al. invented a new flotation process using 2# oil, water glass, and oleic acid to obtain a better separation effect by adjusting the dosage of this combination and combining it with better process conditions [16]. To separate magnesite from quartz in reverse flotation, a cationic collector, such as dodecylamine (DDA), is typically used, which can be partially adsorbed on the surface of magnesite and change the surface properties of magnesite due to its strong electronegativity [17,18,19] and weak spatial site resistance defects [20]. Liu et al. [21] investigated the flotation performance of magnesite and quartz using dodecylamine, N-dodecyl isopropanolamine (DIPA) containing one isopropanol substituent, or N, N-dodecyl diisopropylamine (DDIPA) containing two isopropanol groups. The results showed that introducing isopropanol substituents in DDA weakened the electrochemical properties of the polar groups and increased the cross-sectional size, thereby reducing the electrostatic effect and enhancing the spatial location barrier effect. This improved the selectivity of the agent and increased the flotation efficiency. Yu et al. [22] conducted a reverse flotation test on a low-grade magnesite ore, where they utilized a new collector known as LKD (fatty acid oxidized by ozone), resulting in a final concentrate with 97.10% MgO content. The obtained concentrate’s MgO grade was significantly higher than that obtained with DDA utilizing the same process and chemical conditions. In carbonate ore flotation, the use of the direct flotation method with oleic acid and oleate as collectors is widespread. Sun et al. [23] studied the influence of modifiers on the separation of cryptocrystalline magnesite and dolomite. The results show that sodium hexametaphosphate can better inhibit dolomite and realize the separation of Ca and Mg followed by the inhibitory effect of water glass and sodium fluorosilicate.

It is well known that mineral particles in the range of approximately 20–150 μm are floated quickly with a high flotation recovery; the recovery drops dramatically for particles outside this range. Generally, the reduced recovery of +150 μm particles is attributed to the increased detachment of the particles from the particle–bubble aggregates when subjected to the turbulent flow field. For the −20 µm fine and ultrafine particles, the inertia forces are diminishing while the effects of viscous drag start to dominate; however, there are still knowledge gaps in the fundamental understanding of micro-processes in flotation.

**Figure 1:**
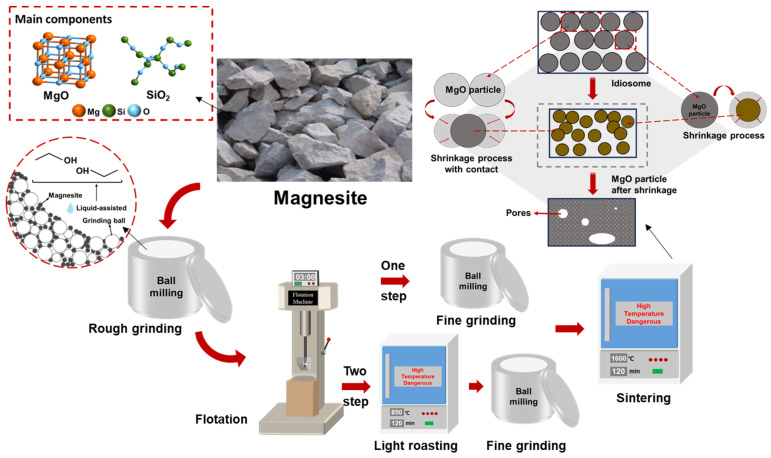
Preparation process of sintered magnesium.

After flotation and purification, magnesite is typically calcined to produce sintered magnesite, which is then used as a refractory raw material. Currently, a two-step calcination process is commonly used to prepare sintered magnesia from magnesite. First, the crushed magnesite is purified by flotation and then lightly roasted to prepare calcined magnesia, which is further ground and then calcined to produce sintered magnesia. If magnesite is ground to a certain fineness, purified by flotation, and calcined to produce sintered magnesia directly after flotation and purification, using a one-step calcination process will not only greatly improve production efficiency but also save a significant amount of energy. The specific technical route is illustrated in Figure 1.

## 2. Materials and Methods

### 2.1. Materials

The magnesite raw material was extracted from Haicheng City, Liaoning Province, China with the chemical composition as shown in Table 1.

**Figure 2:**
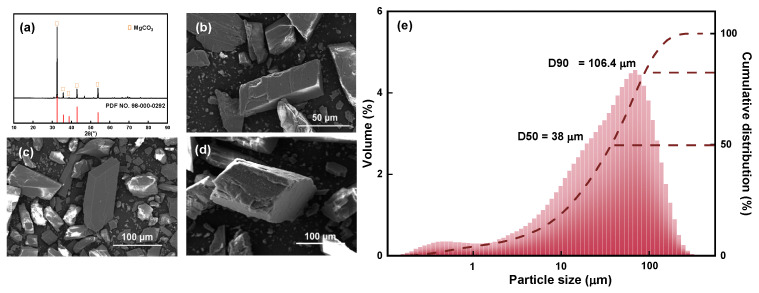
(**a**) XRD pattern, (**b**–**d**) SEM images, and (**e**) particle size and cumulative distribution of magnesite raw material.

The XRD pattern, SEM images, and particle size and cumulative distribution of the magnesite raw material are shown in Figure 2.

The XRD pattern shown in Figure 2a indicates that the main chemical component of magnesite is MgCO_3_, and the main diffraction peak is relatively sharp. Combined with the chemical composition analysis, it can be observed that the magnesite sample used is of high purity and meets the test requirements; the sample can be directly used without further purification. Figure 2b–d are SEM images of magnesite raw ore at different magnifications. The single particles are regular and complete with no agglomerate adsorption state between particles, and the initial particle size is 38 μm (Figure 2e), which is evenly distributed and suitable for further fine grinding.

The reagent information used in the flotation process is shown in Table 2.

AR represents analytical reagent and CP represents chemical pure. The process of reverse flotation involves the use of dodecylamine and sodium polyacrylate solution as the collector and inhibitor, respectively. The dodecylamine solution was prepared by mixing hydrochloric acid and dodecylamine in a 1:1 molar ratio. In the direct flotation process, sodium oleate was utilized as the collector. To regulate pH, a 1% mass concentration of NaOH solution was employed.

### 2.2. Grinding and Flotation

The raw magnesite was placed into a planetary ball mill jar and mixed with ethanol as a solvent for ball milling using the wet method. Following the fine grinding, the ethanol suspension was poured into trays and then dried in an oven at 80 °C.

**Figure 3:**
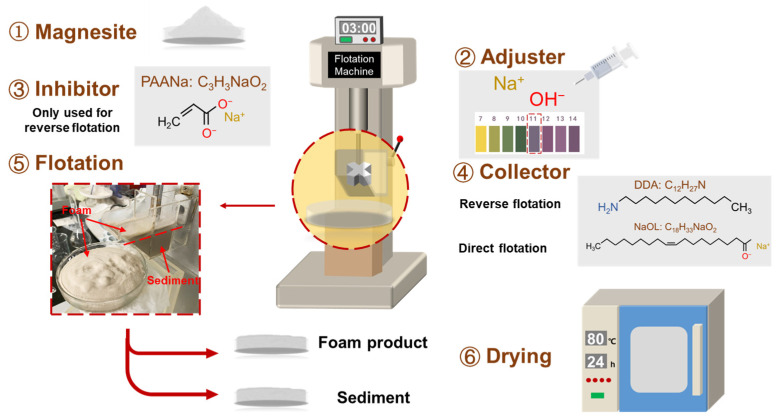
The flotation process of magnesite.

The experiment involved a single mineral flotation test using a laboratory XFG self-aeration flotation machine (Changchun Exploration Machinery Factory, Changchun, China) with the impeller speed fixed at 1992 r/min. Five grams of magnesite was added to the flotation tank, which contained 35 mL of deionized water. The mixture was then stirred for 3 min to form a slurry. All flotation tests were conducted at room temperature. The flotation products were collected and dried sufficiently, and the flotation recovery was calculated using Equation (1).

(1) $$\varepsilon_{i}=\frac{m_{i}\times \beta_{i}}{m_{t}\times \omega }\times 100\%,$$

where *ε_i_* is the recovery of the target element minerals (%), *m_i_* and *m_t_* are the weight of the target element in the mineral and raw ore (*m*), respectively, *β_i_* is the grade of the target element (%), and *ω* is the purity of magnesite (%). The flotation process is shown in Figure 3.

### 2.3. Characterization

The particle size distribution of the specimens was determined using a Mastersizer 3000 laser particle size analyzer (Malvern Instruments Ltd., Malvern, UK); the particle morphology and element distributions of the specimens were observed and analyzed using a scanning electron microscope (SEM, Apreo 2, Thermo Scientific, Waltham, MA, USA) equipped with energy-dispersive X-ray spectroscopy (EDS). The phase composition of the magnesite was determined via X-ray diffraction (XRD, Philips PW3040/60, Amsterdam, The Netherlands) measurements using a Cu Kα (λ = 0.154056 nm) radiation source operated at 40 kV and 40 mA. The angular scan range was 2θ = 10−70°, and the scan rate was 5°/min.

## 3. Results and Discussion

### 3.1. Particle Distribution State of Magnesite after Fine Grinding

**Figure 4:**
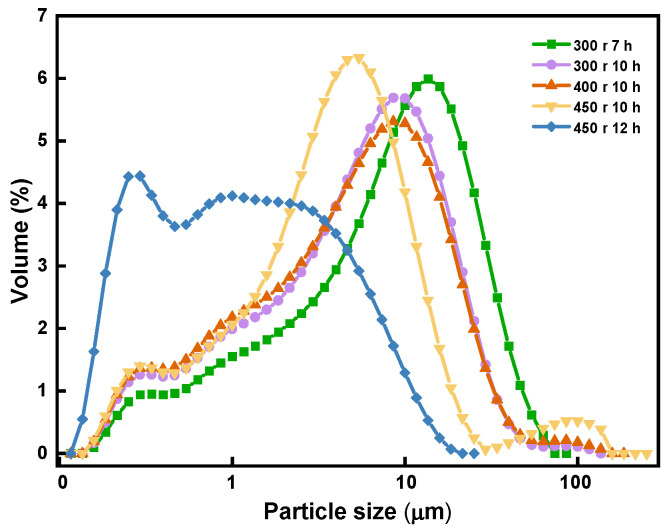
Correlation between particle size and grinding time.

Figure 4 depicts the correlation between the size of the magnesite particles and their grinding time.

As shown in the figure, the milling duration resulted in a decrease in the size of the magnesite particles. When the magnesite was ground to a degree where high-density sintered magnesia can be obtained through one-step sintering using a ball mill speed of 450 r, the grinding time ought to be limited to approximately 12 h.

**Figure 5:**
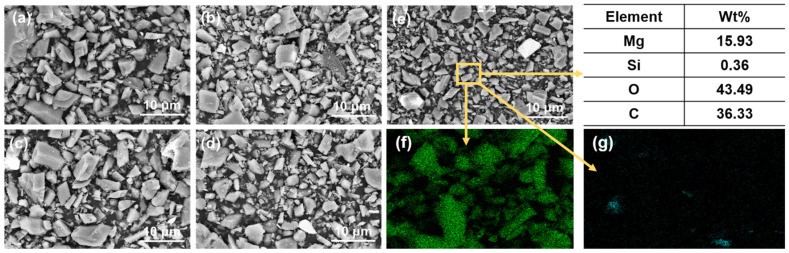
SEM images of magnesites with different particle sizes: (**a**) 300 r 7 h; (**b**) 300 r 10 h; (**c**) 400 r 10 h; (**d**) 450 r 10 h; (**e**) 450 r 12 h; (**f**) the distribution of Mg elements; (**g**) the distribution of Si elements.

Figure 5 presents the SEM images and EDS results of the magnesites ground for different lengths of time.

Figure 5a–e correspond to the five samples shown in Figure 5, while Figure 5f,g display the distribution of Mg and Si elements in magnesite with D50 of 2.83 μm, respectively. As shown in the figures, there is no agglomeration of magnesite particles in different particle distributions, and the MgO and SiO_2_ distributions are relatively independent and uniform, indicating a high degree of dissociation. These findings suggest that the magnesite samples are suitable for single mineral flotation.

### 3.2. Magnesite Concentrate Grade and Recovery

**Figure 6:**
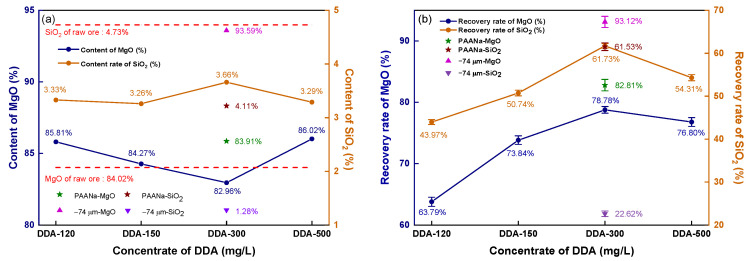
Reverse flotation concentrate’s grade and recovery: (**a**) concentrate grade; (**b**) recovery.

Figure 6 depicts the impact of DDA concentration on the reverse flotation of magnesite.

The contents of MgO and SiO_2_ in the raw ore are represented by red dashed lines in Figure 6a. The flotation concentrate shows a lower SiO_2_ content than the raw ore (4.73%), ranging from 3.26 to 3.66% as the DDA concentration increases, while the MgO content is higher than that of the raw ore (84.02%) at all levels except for 300 mg/L DDA. Unground magnesite floated at a 300 mg/L DDA concentration yield of concentrate with 93.59% MgO and only 1.28% SiO_2_. To strengthen the collector effect, an inhibitor, sodium polyacrylate (PAANa), was added at 150 mg/L based on 300 mg/L DDA, which inhibits MgO from entering the foam layer, enhancing the recovery rate of MgO and resulting in an 83.91% MgO content and 4.11% SiO_2_ content.

As shown in Figure 6b, when the DDA concentration increased from 120 mg/L to 300 mg/L, the recovery rate of MgO significantly increased, and the recovery rate reached the maximum value of 78.78% at the 300 mg/L concentration. However, increasing the DDA concentration further causes the recovery rate to decline. The recovery rate of magnesite flotation with 300 mg/L DDA without fine grinding reaches 93.12%. By analyzing the recovery rate of SiO_2_ in the concentrate, it can be observed that the recovery effect of SiO_2_ was very similar to that of MgO. When the DDA concentration was increased to 300 mg/L, the recovery rate of SiO_2_ reached a peak at 61.73%. A further increase in the DDA concentration resulted in an increase in the recovery rates of MgO and SiO_2_, indicating that DDA has a similar impact in collecting MgO and SiO_2_. Interestingly, even with the addition of PAANa, the recovery rate of MgO in the concentrate significantly increased to 82.81%, while the recovery rate of SiO_2_ still reached a noticeable 61.53%, which is similar to the recovery rate of SiO_2_ without inhibition. If the inhibitor and collector only affect one of the minerals, then an increase in the recovery rate of MgO would be accompanied by a decrease in the recovery rate of SiO_2_. As evidenced by the recovered rates, there was a direct correlation between the recovery rates of MgO and SiO_2_. As MgO recovery increases, SiO_2_ recovery also increases, and vice versa. If the content of these minerals follows the same trend as the recovery rate, then as long as the MgO content and recovery rate in the mineral are sufficiently high, it can still be used as the raw material for high-density sintered magnesia. Figure 5 shows the grade and recovery rate of the magnesite concentrate obtained by direct flotation with sodium oleate (NaOL) as a collector.

**Figure 7:**
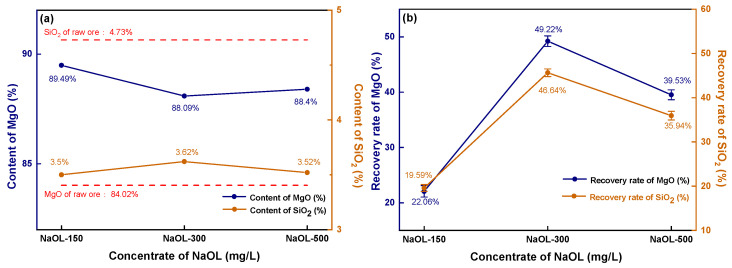
Direct flotation concentrate’s grade and recovery: (**a**) concentrate grade; (**b**) recovery.

As observed in Figure 7a, the magnesite concentrate obtained through direct flotation has an extremely high MgO content, reaching up to 89.49% when the NaOL concentration is 150 mg/L.

With an increase in NaOL concentration, the MgO content becomes higher than that of the original ore (84.02%). The SiO_2_ content fluctuates within the range of 3.50–3.62%, which is significantly lower than the SiO_2_ content (4.73%) in the raw ore. However, the recovery rate of direct flotation magnesite is much lower than that of reverse flotation magnesite. As shown in Figure 7b, the MgO recovery rate is less than 20% when the NaOL concentration is 150 mg/L. Even with an increase in NaOL concentration resulting in a peak, the recovery rate of MgO is still less than 50%, which greatly reduces the recovery efficiency of magnesite. After comparing the experimental results of direct and reverse flotation of magnesite, it can be concluded that reverse flotation is an effective method for desilication and the removal of impurities of magnesite. Under constant reagent concentration, the recovery rate and MgO content without fine grinding are much higher than those of magnesite with fine grinding. Therefore, the reason why the flotation desilication of fine magnesite cannot achieve the desired results is due to the agglomeration between MgO and SiO_2_ particles caused by superfine magnesite particles. During the reverse flotation process, the flotation agent cannot target a single element but plays a collecting or inhibiting role on the whole agglomerate, resulting in the recovery rate of MgO in the concentrate never reaching an ideal state.

While discussing direct flotation, although the MgO content is enhanced, the weight of fine particles is much greater than that of dispersed particles after the agglomeration. As a result, the minerals entering the foam layer decrease, which negatively impacts the recovery rate of magnesite. It is evident that the size of the magnesite particles is directly linked to the flotation recovery rate. Specifically, when the average magnesite particle size is 2.83 μm, agglomeration or entrapment between the fine particles arises during flotation, making it challenging to distinguish magnesia containing mineral particles from silicon containing mineral particles, thereby affecting the flotation result.

### 3.3. Presence State of the Silica Phase of Magnesite Concentrate

**Figure 8:**
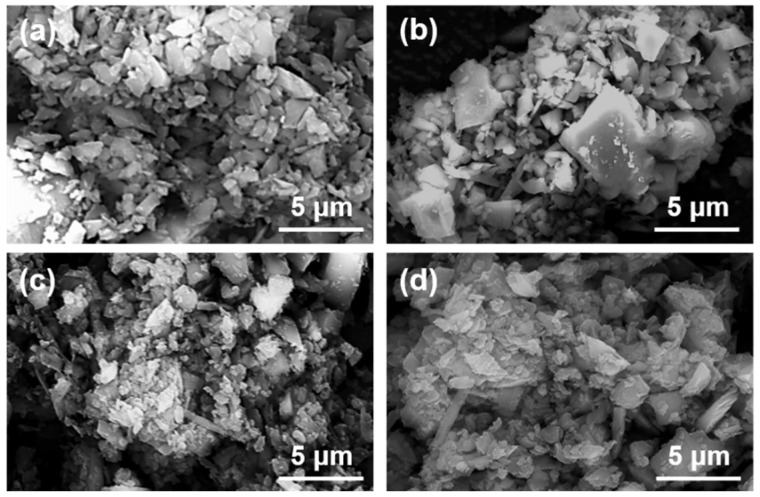
SEM images of magnesite concentrate under different DDA concentrations: (**a**) DDA = 120 mg/L; (**b**) DDA = 150 mg/L; (**c**) DDA = 300 mg/L; (**d**) DDA = 500 mg/L.

Figure 8 shows the micrographs of magnesite particles following flotation with collectors of varying concentrations.

**Figure 9:**
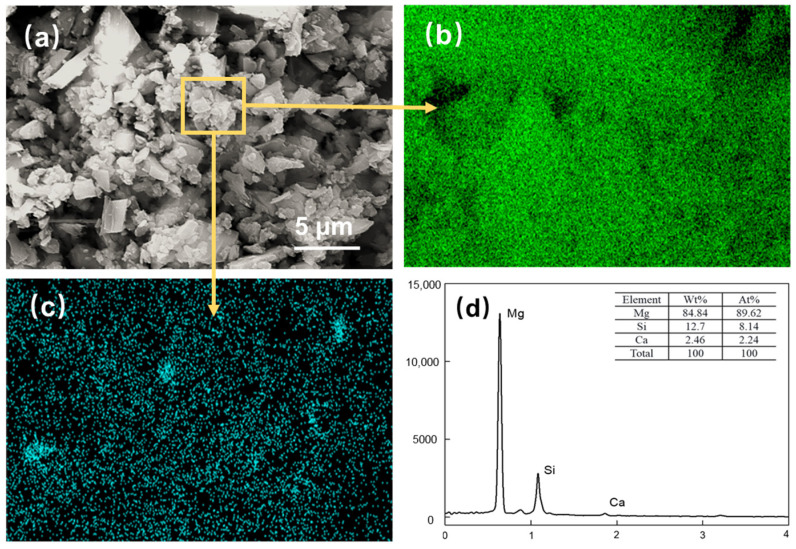
Magnesite concentrates after DDA (300 mg/L) and PAANa (150 mg/L) flotation: (**a**) SEM image; (**b**) EDS mapping for magnesium element; (**c**) EDS mapping for silicon element; (**d**) elemental content.

The magnesite used for flotation was ground to a D50 of 2.83 μm. As can be observed in Figure 8, the large particles are surrounded by small particles of minerals, forming mineral aggregates with larger particle sizes. The number of individual mineral particles is minimal, and an apparent agglomeration structure is formed between the minerals. Based on this, the samples with the addition of inhibitors were further analyzed as shown in Figure 9.

**Figure 10:**
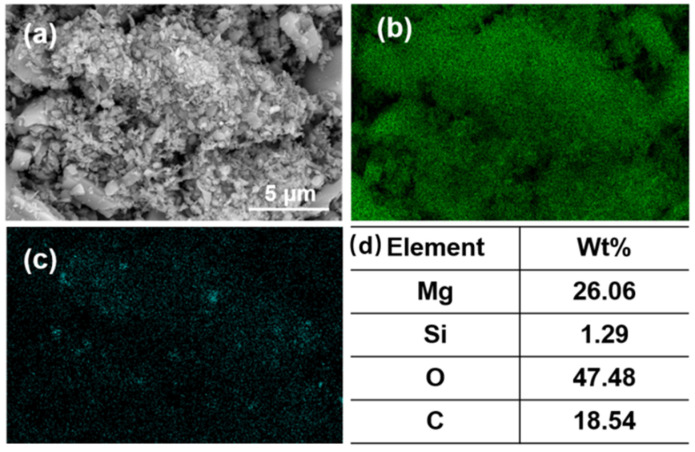
(**a**) SEM image; (**b**) EDS mapping for magnesium element, (**c**) EDS mapping for silicon element, and (**d**) elemental content of fine particles (<1 μm) in the agglomerates.

Figure 9a shows the SEM image of the flotation concentrate of 150 mg/L PAANa added at 300 mg/L DDA; the elemental contents of magnesium and silicon within this interface were scanned, and a certain area in the image was selected for compositional content analysis as shown in Figure 9d. The distribution of elements in the image was uniform with a large amount of silicon appearing in a few areas. The magnesium content in the square frame was 84.84%, while the silicon content was up to 12.7%. Additionally, a significant number of agglomerates consisting of fine particles with particle sizes less than 1 μm exist in the magnesite flotation, as shown in Figure 10.

According to the elemental analysis of the agglomerates, the fine particles are composed of minerals containing MgO and SiO_2_. The finer the mineral particle size, the higher the SiO_2_ content, which increased from 0.36% before flotation to 1.29% after flotation. This situation is due to the fact that the particle size of magnesite was too fine. The smaller the radius of the mineral particles, the lower the energy of mutual repulsion between the particles. As a result, MgO and SiO_2_ particles easily agglomerate in the flotation process. The function of the collector has changed from solely targeting SiO_2_ to a mineral agglomerate of MgO and SiO_2_. Thus, the increase in SiO_2_ or decrease in MgO content has the same trend.

**Figure 11:**
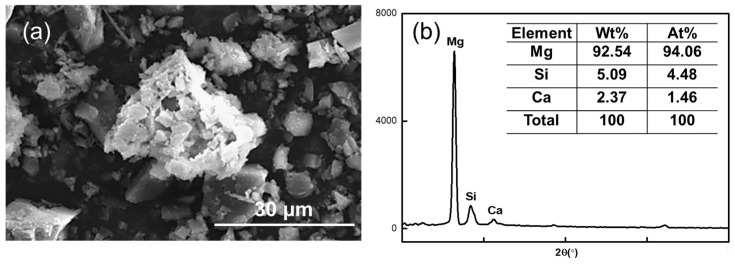
(**a**) SEM image of direct flotation magnesite concentrate at NaOL concentration of 150 mg/L and (**b**) elemental content in the agglomerates.

Figure 11 shows the SEM image of the magnesite concentrate in direct flotation when the NaOL concentration was 150 mg/L.

The MgO content of the agglomerate in the image is 94.06%, the SiO_2_ content is 4.48%, and the widest diameter can reach 32.73 μm, which is 11 times the diameter of magnesite particles after grinding. This further confirms that the magnesite after grinding is susceptible to agglomeration. Thus, the quality of the minerals is too large, and the adhesion degree with air bubbles is reduced during the positive flotation process, which affects the recovery rate of magnesite.

### 3.4. Effect of Chemical Properties on Flotation

**Figure 12:**
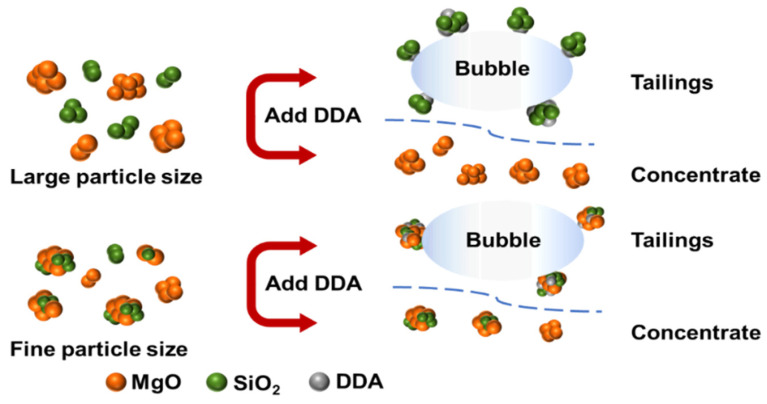
Mechanism of the effect of particle size on magnesite flotation.

Figure 12 illustrates a plot of the mechanism of the effect of particle size on the desilication of magnesite by reverse flotation.

The flotation process involves the collision and adhesion between bubbles and particles, which belongs to fluid dynamics. In flotation slurry, the behavior and state of microfine grained minerals are still explained by the basic principles of colloid chemistry due to the small size and mass, large specific surface area, and surface charge of the ore. The theory of colloidal particle stability in dispersion systems, namely the classical DLVO theory, studied the stability of charged colloidal particles [24], examining their stability and agglomeration from the perspective of the interaction between repulsive and suction potential energy between colloidal particles. This theory suggests that there are two types of interaction forces between charged colloidal particles: electrostatic repulsion of the double electric layer and van der Waals between particles [25]. Their interaction determines the coalescence and dispersion behavior between mineral particles in low concentration electrolyte slurry, as shown in Equation (2).

(2) $$V_{T}^{D}\left(H\right)=V_{vdW}\left(H\right)+V_{edl}\left(H\right),$$

where $V_{T}^{D}\left(H\right)\;$ is the total interaction energy, *V_vdW_*(*H*) is the van der Waals force interaction energy, and *V_edl_*(*H*) is the double layer force interaction energy.

Van der Waals force widely exists between substances, colloids, and particles. When there is a significant difference in particle diameter, Equation (3) is frequently used for the calculation.

(3) $$V_{w}=-\frac{A_{132}R_{1}}{6H},$$

However, when the difference in particle diameter is small, it may be more appropriate to use Equation (4).

(4) $$V_{W}=-\frac{A_{132}}{6H}\frac{R_{1}R_{2}}{R_{1}+R_{2}},$$

where *R*_1_, *R*_2_ is the radius of the spherical particles, *H* is the interaction distance, and *A*_132_ is the Hamaker constant for the interaction of particle 1 and particle 2 in medium 3, calculated as shown in Equation (5).

(5) $$A_{132}\approx \left(\sqrt{A_{11}}-\sqrt{A_{33}}\right)\left(\sqrt{A_{22}}-\sqrt{A_{33}}\right),$$

where *A*_11_, *A*_22_, and *A*_33_ are Hamaker constants for particle 1, particle 2, and medium 3 in a vacuum, respectively. Since water is a polar molecule, the intermolecular motion also includes orientation and induction forces, so Equation (5) is modified to the following Equation:

(6) $$A_{132}=A_{132}^{0}+A_{132}^{\xi },$$

(7) $$A_{132}^{0}=\frac{3}{4}k_{B}T\left(\frac{\varepsilon_{1}-\varepsilon_{3}}{\varepsilon_{1}+\varepsilon_{3}}\right)\left(\frac{\varepsilon_{2}-\varepsilon_{3}}{\varepsilon_{2}+\varepsilon_{3}}\right),$$

(8) $$A_{132}^{\xi }=\frac{3h_{\nu e}}{8\sqrt{2}}\frac{\left(n_{1}^{2}-n_{3}^{2}\right)\left(n_{2}^{2}-n_{3}^{2}\right)}{{\left(n_{1}^{2}+n_{3}^{2}\right)}^{1/2}{\left(n_{2}^{2}+n_{3}^{2}\right)}^{1/2}\left[{\left(n_{1}^{2}+n_{3}^{2}\right)}^{1/2}+{\left(n_{2}^{2}+n_{3}^{2}\right)}^{1/2}\right]},$$

where *k_B_* is Boltzmann’s constant, taken as 1.38 × 10^−23^ J/K, *T* is the absolute temperature (K); *ε*_1_, *ε*_2_, *ε*_3_ and *n*_1_, *n*_2_, *n*_3_ are the dielectric constant and refractive index of particle 1, particle 2, and medium 3, respectively, and the values are shown in Table 3; h is Planck’s constant, and its value is 6.626 × 10^−34^ J/s; ν_e_ is the primary electron absorption frequency in ultraviolet light with the value of 3 × 10^15^ s^−1^.

The energy that the double electrical layer exerts is typically calculated using the Hogg–Healy–Fuerstenau (HHF) equation. Equations (9)–(11) depict the HHF equation for two distinct particles in close proximity.

(9) $$V_{edl}\left(H\right)=\frac{\pi \varepsilon_{0}\varepsilon_{r}R_{1}R_{2}}{R_{1}+R_{2}}\left(\varphi_{1}^{2}+\varphi_{2}^{2}\right)\left(\frac{2\varphi_{1}\varphi_{2}}{\varphi_{1}^{2}+\varphi_{2}^{2}}p+q\right),$$

(10) $$p=\ln\left[\frac{1+\exp\left(-\kappa H\right)}{1-\exp\left(-\kappa H\right)}\right],$$

(11) q=ln[1−exp(−2κH)],

where *ε*_0_ is the absolute permittivity in a vacuum of 8.854 × 10^−12^ F/m, *ε_r_* is the absolute permittivity of the dispersion, *ε_r_* = 78.36 F/m for aqueous media, *φ*_1_ and *φ*_2_ are usually the surface potential of the mineral, which is generally replaced by the zeta potential [26] in V; H is the distance between two particles in nm; *κ*^−1^ is the Debye length, and for 1:1 type electrolyte, *κ*^−1^ = 0.304 × C^−1/2^ nm^−1^, and *κ*^−1^ = 9.6 nm for a KCl concentration of 10^−3^ mol/L.

The classical DLVO theory can be applied to analyze the coalescence or dispersion behavior of MgO and SiO_2_ particles in the flotation process. According to the theory, when the total interaction energy $V_{T}^{D}\left(H\right)$ is greater than zero, the particles repel each other and remain dispersed. Conversely, when $V_{T}^{D}\left(H\right)$ is less than zero, the particles attract each other and are coalesced.

The Equation (9) demonstrates that the total interaction energy $V_{T}^{D}\left(H\right)$ is positively correlated with the mineral particle size R. As the particle size decreases, the double electrical layer repulsion and van der Waals force decrease accordingly, resulting in a lower energy barrier for coalescence between SiO_2_ particles. Using calculations, the total interaction energy $V_{T}^{D}\left(H\right)$ between MgO and SiO_2_ was found to be −8.16 × 10^−7^ when the mineral particle size was D50 = 2.83 μm. Since this value is negative, it indicates a cohesive state between the particles and suggests that agglomeration is the primary reason for the poor flotation recovery of fine particles.

## 4. Conclusions

The effect of mineral size on the desilication of magnesite through grinding and flotation was studied. The mechanism of flotation desilication of microfine magnesite was investigated by combining microstructure and chemical properties, and the following conclusions were obtained:

1. Dodecylamine was found to be the most effective collector for the reverse flotation of microfine magnesite with a particle size of D50 = 2.83 μm. The best collection effect of 78.78% was achieved with a DDA concentration of 300 mg/L and slurry pH of 10. However, the recovery of magnesite without fine grinding was as high as 93.12% with the same concentration of collector. Further addition of the inhibitor sodium polyacrylate (PAANa) at 150 mg/L increased the recovery of MgO in the concentrate to 82.81%, an increase in recovery of 5.16%. Sodium oleate (NaOL) was found to be a good direct flotation collector, and a concentrate with a high MgO content of 89.49% was obtained, while the recovery was less than 50% and independent of the agent concentration. The poor flotation effect of magnesite was attributed to the fine particle size, which caused agglomeration of MgO and SiO_2_ particles in the flotation process and hindered the flotation agent from acting on single element minerals.
2. According to the classical DLVO theory and calculation, the total interaction energy $V_{T}^{D}\left(H\right)$ = −8.16 × 10^−7^ between MgO particles and SiO_2_ particles was smaller than zero in the cohesive state when the mineral particle size was 2.83 μm. This indicated that agglomeration occurred between the particles. Therefore, interparticle agglomeration of fine particles was the main cause of the low flotation recovery.

## Figures and Tables

**Table 1:** Chemical composition of magnesite.

Component	MgO	SiO_2_	CaO	Al_2_O_3_	Fe_2_O_3_
Content (%)	84.02	4.73	2.92	0.36	5.60

**Table 2:** Information on the reagents used for flotation.

Reagents	Chemical Formula	Specification	Manufacturer
Dodecylamine	C_12_H_27_N	CP	Sinopharm Chemical Reagent Co., Ltd., Shenyang, China.
Sodium polyacrylate	C_3_H_3_NaO_2_	CP	Sinopharm Chemical Reagent Co., Ltd. Shenyang, China.
Sodium oleate	C_18_H_33_NaO_2_	CP	Sinopharm Chemical Reagent Co., Ltd. Shenyang, China.
Sodium hydroxide	NaOH	AR	Sinopharm Chemical Reagent Co., Ltd. Shenyang, China.

**Table 3:** Dielectric constants and refractive indices of materials.

	MgO	SiO_2_	Water
Dielectric constants (F/m)	9.65	3.9	78.36
Refractive index	1.16	1.54	1.33

## Data Availability

Not applicable.
